# Effects of functionalized multi-walled carbon nanotubes on toxicity and bioaccumulation of lead in *Daphnia magna*

**DOI:** 10.1371/journal.pone.0194935

**Published:** 2018-03-29

**Authors:** Min-Hee Jang, Yu Sik Hwang

**Affiliations:** Future Environmental Research Center, Korea Institute of Toxicology, Jinju, Gyeongsangnam-do, Republic of Korea; VIT University, INDIA

## Abstract

Increased production of carbon nanotubes (CNTs) and their widespread application in industrial and consumer products have led to a rise in the release of CNTs into the aquatic environment. CNTs have a very strong adsorption affinity for various environmental contaminants; therefore, they may also influence the toxic effects of other pollutants, such as toxic metals. In this study, the effect of two different functionalized carbon nanotubes, carboxylate and polyethyleneimine modified multi-walled carbon nanotubes (C-MWCNTs and N-MWCNT, respectively) on lead toxicity and bioaccumulation was investigated with a freshwater zooplankton *Daphnia magna*. The acute toxicity results indicate that the different surface properties of the two types of MWCNTs have different effects on lead toxicity to *D*. *magna*. The negatively charged C-MWCNT showed a notable decrease in lead toxicity (LC_50_ value increased from 0.15 mg L^-1^ to 1.08 mg L^-1^ in the presence of 10 mg L^-1^ C-MWCNT), whereas the positively charged N-MWCNT had only a slight effect on lead toxicity (LC_50_ value increased from 0.15 mg L^-1^ to 0.16 mg L^-1^ in the presence of 10 mg L^-1^ N-MWCNT). The decrease of lead toxicity was related with the reduced bioavailability of free metal form (Pb^2+^) caused by greater adsorption of lead onto the MWCNTs. The present study suggests that there is a need to consider carefully the complex interaction of CNTs with toxic metals in future ecotoxicological studies.

## Introduction

Increased production and wider application of carbon nanotubes (CNTs) have led inevitably to their entry into the aquatic environment, and this release is likely to bring about unexpected hazards in various organisms [1, 2]. Toxic mechanisms of CNTs toward a variety of organism species have been studied in many *in vivo* and *in vitro* tests [3–5]. Although the number of studies investigating the toxic effects of CNTs alone could give invaluable information, in the real environment CNTs actually coexist with other toxic compounds. Furthermore, CNTs exhibit a very strong adsorption affinity for various environmental contaminants [6–8]; thus, they may also influence the fate of other aquatic pollutants that surround them, such as toxic metals. Interactions of CNTs with other toxic compounds may influence the transport of toxic materials and further affect their bioavailability and toxicity in the environment. However, there is a critical gap in our knowledge on the mechanisms involved in the interaction of CNTs with other pollutants and the effects of these interactions on the behavior and toxicity of both the CNTs and the other pollutants.

Functionalized carbon nanotubes (F-CNTs) are considered promising composite materials for industrial applications because of ease of dispersion and improvement of functionality for specific applications [9–11]. Functionalization of CNTs can modify the physicochemical properties of pristine CNTs as well as their environmental fate and toxicities. Several studies showed that the functionalization of CNTs generally increases their stability in aqueous media. Lee et al. [12] measured the dispersion stability of pristine and surface modified CNTs, and the results revealed that CNTs modified with carboxylic groups greatly enhanced dispersibility in polar solvents. Uo et al. [13] have shown that the functionalization of CNTs prevents agglomeration and alters the toxic effects on various organisms. Also surface functionalization has an effect on the interaction of CNTs with other pollutants. Tofighy et al. [14] demonstrated that adsorption capacities of metal ions onto CNTs strongly depend on their surface acidity including functional groups. Park et al. [15] showed that the oxygen of the functional group directly interacts with metal ions and thus plays a key role in the adsorption process. Therefore, more investigations are needed into the adsorption properties of F-CNTs with hazardous pollutants and how these interactions influence the environmental risks.

Lead is a ubiquitous and common pollutant and ranks as one of the most hazardous heavy metals [16]. Exposure to lead may cause various types of damage in brain, kidney, nervous system, and reproductive system [17]. The behavior (i.e., mobility, bioavailability, and toxic effects) of lead is closely related with the form in which it exists in the aquatic environment. The free metal form (Pb^2+^) is considered to be the most bioavailable and toxic species. Altindag et al. [18] investigated the acute toxicity of lead nitrate to *Daphnia magna* and found the 24 h EC_50_ of lead is 0.51 mg L^-1^. However, the acute toxicity of lead in aquatic organisms could decrease under the influence of various environmental conditions (i.e., increase in hardness and alkalinity, low pH, and complexation of lead by natural organic matter (NOM)) [19, 20]. Especially, NOM has various metal-binding functional groups such as carboxyl, phenolic nitrogen, and sulfur containing groups and these can easily bind with Pb^2+^ in water. In a similar way, F-CNTs could affect the bioavailability and acute toxicity of lead by influencing the forms of lead that exist in the aquatic environment.

In this work, we tested the hypothesis that different functionalized multi-walled carbon nanotubes (F-MWCNTs) have different adsorption capacities and, hence, affect the risk of toxic compounds surrounding them. For this purpose, two different functionalized carbon nanotubes, carboxylate and polyethyleneimine modified multi-walled carbon nanotubes (C-MWCNT and N-MWCNT, respectively) were employed to compare the effects of F-MWCNTs on lead toxicity to *Daphnia magna*. In addition, the mechanisms involved in the lead adsorption onto F-MWCNTs were thoroughly examined to investigate how F-MWCNTs influence the bioavailability and environmental risk of lead.

## Materials & methods

### Test organism & materials

*Daphnia magna*, a freshwater invertebrate, was used as a test organism. Daphnids were cultured in the laboratory in Elendt M4 medium at 20 ± 2°C with a 16 h light and 8 h dark photoperiod. A green alga, *Pseudokirchneriella subcapitata*, and a combination of yeast, Cerophyll, and trout chow (YCT) were supplied every day. To avoid the potential influence of high ionic strength on the stability of F-MWCNTs, 10-time diluted M4 medium was used in all experiments. In addition, ethylenediaminetetra acetic acid (EDTA) was excluded from the experimental medium because it could be a strong metal binding ligand.

Two different functionalized MWCNTs (purity greater than 95%) were used in this study. Carboxylate (COOH) and polyethyleneimine (PEI) modified MWCNTs (C-MWCNT and N-MWCNT, respectively) were purchased from NanoLab Inc. (Waltham, MA, USA). These functional groups were selected because of their differences in surface charge. Stock suspensions of F-MWCNTs were prepared by adding 300 mg of F-MWCNTs into 300 mL of deionized water in a 400 mL glass beaker. The mixture was ultra-sonicated for 30 min using a probe sonicator at an intensity of 52 W with 5 s intervals. The stock suspensions were kept at room temperature and were weakly sonicated before use.

Morphology of F-MWCNTs was analyzed using a transmission electron microscope (TEM, Tecnai F30ST (300 kV), FEI, USA). The specific surface area was examined by a micropore physisorption analyzer (ASAP-2020M, Micrometritics Instrument, USA). Hydrodynamic diameter and zeta potential of particles in the water suspensions were measured using a Zetaszer Nano (Nano ZS 90, Malvern Instruments, UK). To characterize the surface charge of F-MWCNTs, electrophoretic mobility was measured using a Zetasizer Nano over a range of pH in 10 mM NaCl. Analysis of surface elements and chemical state of F-MWCNTs was carried out using an X-ray photoelectron spectrometer (XPS, K-Alpha, Thermo Scientific, USA). Metal impurities in F-MWCNTs were measured by an inductive coupled plasma mass spectrometer (ICP-MS, Elan DRC II, PerkinElmer, USA) after aqua regia digestion. In addition, sedimentation characteristics of F-MWCNTs were determined by monitoring the optical absorbance as a function of time with a Turbiscan LAB stability analyzer (Formulaction SA, France).

### Adsorption experiment

Batch adsorption experiment was employed to evaluate the lead adsorption onto F-MWCNTs. The background solution was 0.01 M NaNO_3_ to maintain a stable ionic strength. In addition, 10-time diluted Elendt M4 medium was used as a background solution for interpretation of the changes of lead toxicity in the presence of F-MWCNTs. For the equilibrium isotherm experiment, the variation of lead adsorption with nominal concentration (0.01, 0.1, 0.5, 1, 2, 5, 10, and 20 mg L^-1^ as Pb^2+^) was examined in the presence of 5 mg L^-1^ F-MWCNTs. Based on previous studies [21–23], duration of the equilibrium isotherm experiment was 24 h. After gently shaking for 24 h at 200 rpm, samples were centrifuged using an Amicon Ultra Centrifugal filter (30 KD) at 4000 rpm for 30 min to separate the particles and dissolved metal. After removing the MWCNTs from solution, the amount of adsorbed lead was calculated using the following equation: *q*_*e*_ *= (C*_*0*_*-C*_*e*_*)V/w*, where *q*_*e*_ is the equilibrium adsorption capacity (mg g^-1^), *C*_*0*_ is the initial lead concentration (mg L^-1^), *C*_*e*_ is the equilibrium lead concentration in background solution (mg L^-1^), *V* is the volume of the solution (L), and *w* is the mass of F-MWCNTs (g). All experiments were carried out in triplicate, and the results are presented as the average measurements of the runs with standard deviations.

### Acute toxicity

A 48 h static non-renewal test was conducted to investigate how F-MWCNTs affect the toxicity of lead to *D*. *magna*. Test solutions were pre-equilibrated one day in advance in a similar method to that performed in the adsorption experiment above. In addition, 10-time diluted M4 medium without EDTA was used as a base of the toxicity media. The exposure concentrations of F-MWCNTs were below 20 mg L^-1^, which did not show 48 h acute toxicity in *D*. *magna*. The test concentrations of lead were 0.01–2.4 mg L^-1^ as Pb^2+^, which were the selected concentrations for efficiently observing an acute effect in the preliminary test. Five neonates (6–24 h old) were placed in a 50 mL polycarbonate bottle containing 40 mL of test solution. After 48 h of exposure, mortality was observed to determine the LC_50_ values. Daphnids were deprived of food for the duration of exposure to avoid adsorption of test materials (i.e., F-MWCNT, lead adsorbed onto F-MWCNTs, and free lead) on the food. As pH level is an important factor influencing the toxicity of lead via the changes of lead species [24, 25], all the experiments were carried out at about pH 6.3 ± 0.2. The LC_50_ values and 95% confidence intervals (CIs) were calculated by the Spearman-Karber method using the CETIS program (1.8.0.9 Version, Tidepool Scientific Software, USA) and quadruplicate samples were used for each condition.

### Accumulation experiments

To identify the changes in the bioaccumulation of lead in *D*. *magna* by F-MWCNTs, experiments were conducted with a 24 h uptake period followed by a 72 h depuration period. All test solutions were made one day in advance and left overnight under the same conditions as in the following experiment for equilibration. Test solutions were prepared by mixing 10 μg L^-1^ lead with 0, 1, and 10 mg L^-1^ F-MWCNTs for 24 h (200 rpm, 20°C). Thirteen individual daphnids (7 d old) were exposed to each test media. At each sampling time point (0, 1, 3, 6, 12, and 24 h), daphnids were collected to quantify the accumulated total lead. The daphnids were rinsed more than three times using fresh M4 medium to remove the loosely adsorbed lead from their carapaces. After daphnids were dried at 95°C to measure their dry weight, total lead concentrations were determined. To observe the depuration phase, the residual daphnids were transferred to clean M4 medium. Water changes were conducted every day to avoid re-exposure of test material excreted from daphnids. On hours 1, 2, 4, 10, 24, 48, and 72 after transfer, daphnids were collected and dried at 95°C until they reached a stable weight. To determine the lead concentrations in daphnids, 8 mL of concentrated nitric acid (70%, Junsei) and 2 mL of hydrogen peroxide (30%, Junsei) were added to the dried daphnids, and samples were completely digested for 4 h at 120°C using a heating block. After complete digestion, the acid solution was allowed to evaporate to dryness. Then, the solid phase extract was eluted with 10 mL of ultrapure water. The concentrations of lead in *D*. *magna* were analyzed by an inductively coupled plasma-mass spectrometer (ICP-MS, NexIon 300X, PerkinElmer, USA). The analytical procedures were validated by conducting a lead spike recovery test in each daphnia (1 or 3 ug of lead in daphnia) and a good mass balance (104.0±6.6%) was achieved. Triplicate tests were carried out for the accumulation experiments.

Toxicokinetic analysis was performed on the basis of lead concentration in *D*. *magna* after exposure to lead and F-MWCNTs using a pseudo-first order model. The elimination rate constant (*K*_*e*_) was determined by fitting a nonlinear regression to the equation $C_{t}=C_{0}e^{-k_{e}t}$, where *C*_*0*_ is the concentration of lead in the body at the beginning of experiment, *C*_*t*_ is the concentration at time *t*, and *t* is time (hours). The half-lives (*t*_1/2_) of lead in *D*. *magna* were estimated according to the equation *t*_1/2_ = −*ln* 0.5/*K_e_*.

## Results

### Physicochemical properties of F-MWCNTs

Physicochemical properties of F-MWCNTs are summarized in Table 1 and S1 Fig. In a comparison of TEM images of C-MWCNT and N-MWCNT no distinguishable difference in morphology was revealed. All samples maintained the original hollow tubular structure (S1(A) and S1(C) Fig). The outer diameters of both F-MWCNTs were in the range of 10–20 nm and the lengths reached several micrometers. These values are largely consistent with those reported by the manufacturer. The specific surface areas of the C-MWCNT and N-MWCNT were estimated to be 194.0 and 118.8 m^2^ g^-1^, respectively. Hydrodynamic diameters of C-MWCNT and N-MWCNT in DI water were 186.2 and 443.5 nm, respectively. The dissimilarities in DLS size may be due to the different surface properties (i.e., surface charge). Zeta potentials for C-MWCNT and N-MWCNT were measured as -40.3 and 25.2 mV, respectively. In addition, electrophoretic mobilities (EPM) of the two F-MWCNTs were fully opposite (S2 Fig). The EPM of C-MWCNT was negative across the entire range of pH and point of zero-charge (PZC) was below pH 2.0. Conversely, the EPM of N-MWCNT was positive below pH 7.5 (PZC was pH 7.5).

**Table 1:** Physicochemical characterization of F-MWCNTs.

MWCNTs	Functional groups	Surface Area (BET)	Primary size (TEM)	Hydro-dynamic diameter (DLS)	Zeta potential	Point of zero charge (PZC) ^3)^
		[m^2^/g]	[d:nm / l:um] ^1)^	[nm] ^2)^	[mV] ^2)^	
C-MWCNT	COOH	194.0	15 ± 5 / 1–5	186.2 ± 6.8	-40.3 ± 0.4	< 2.0
N-MWCNT	polyethyleneimine	118.8	15 ± 5 / 1–5	443.5 ± 16.1	25.2 ± 0.6	7.5

^1)^ Diameter (d) and length (l) were reported in accordance with the manufacturer’s specifications

^2)^ Hydrodynamic diameter and zeta potential were measured with 10 mg L^-1^ MWCNTs in DI water.

^3)^ Point of zero charge was measured with 10 mg L^-1^ MWCNTs in 10 mM NaCl.

### Adsorption of lead on F-MWCNTs

**Fig 1:**
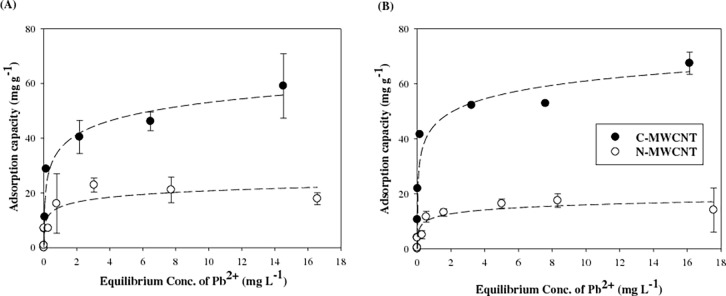
Adsorption isotherms of lead onto F-MWCNTs in (A) 0.01M NaNO_3_ and (B) 10-time diluted M4 medium. Error bars represent the standard deviation.

In a preliminary test using lead solution, the recovery of lead in the filtrate was 96.4±1.4%, indicating that lead sorption onto Amicon Ultra Centrifugal filter (30 KD) did not occur. Previous studies have shown that the rapid adsorption of metals onto CNTs was reached within 30 min and then equilibrated within 4 h [21–23]; therefore, a shaking time of 24 h was selected in this study to ensure equilibrium conditions. The amount of lead adsorbed onto F-MWCNTs in 0.01 M NaNO_3_ and 10-time diluted M4 medium (Fig 1) shows that, in both test conditions, the adsorption capacity of C-MWCNT was much higher than that of N-MWNCT. This result suggests that the negative charge of the C-MWCNT surface could increase the interaction with lead ions and improve the adsorption capacity. Previous studies also have shown that CNTs functionalized with oxygen containing groups dominated the sorption capacity for metals from water [15, 26, 27].

**Fig 2:**
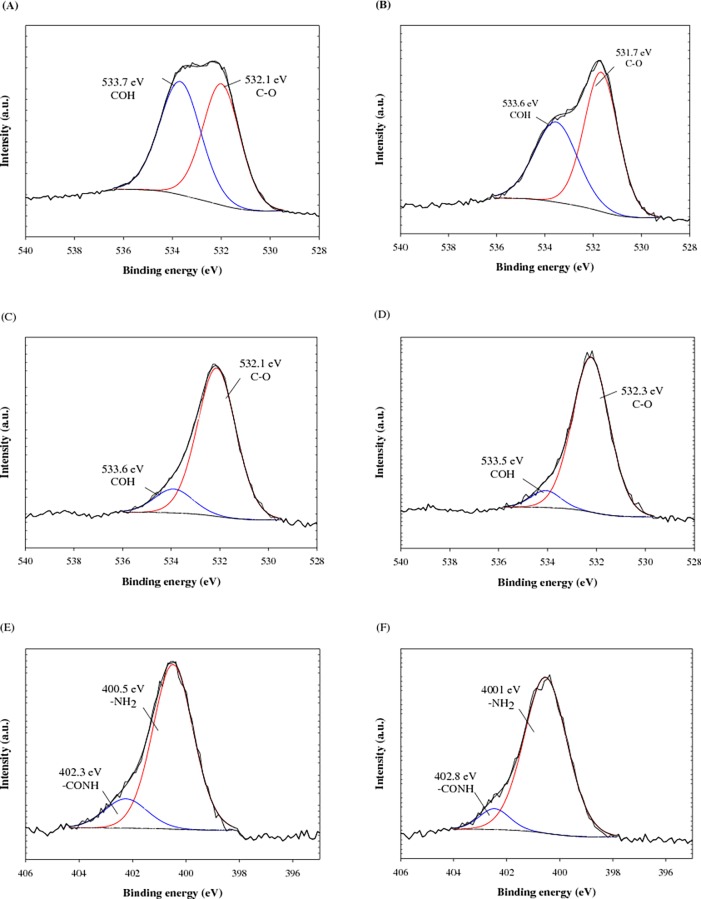
XPS spectra of adsorbent. (A) O1s of C-MWCNT, (B) O1s of C-MWCNT after lead adsorption, (C) O1s of N-MWCNT, (D) O1s of N-MWCNT after lead adsorption, (E) N1s of N-MWCNT, (F) N1s of N-MWCNT after lead adsorption.

**Fig 3:**
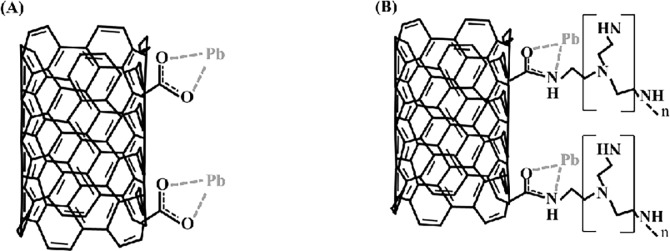
Proposed adsorption mechanisms between lead and F-MWCNTs. (A) C-MWCNT and (B) N-MWCNT.

For further examination of the properties of F-MWCNTs after lead adsorption, the surface binding state and elemental speciation of MWCNTs were analyzed by an XPS. In the wide-scan XPS survey spectra, the binding energies for the C1s, N1s, O1s, and Pb4f were observed at approximately 285.0, 400.2, 532.0, and 138.8 eV, respectively (S3 Fig). Especially, the peak attributable to lead (138.8 eV) was observed after lead adsorption. The O1s spectrum of F-MWCNTs and N1s of N-MWCNT are shown in Fig 2. The O1s peaks of F-MWCNTs can be fitted to two individual component peaks, which are attributed to C = O (BE 532.1 eV) and COH (BE 533.7 eV). After lead adsorption onto the C-MWCNTs, the peak of COH decreased notably, indicating that carboxyl-lead complexes were formed between unoccupied electron orbitals of lead ions and lone pair electrons of oxygen atoms of carboxylate groups (Fig 2(A) and 2(B)) [28, 29]. In the case of N-MWCNT, the peak of COH was relatively smaller than that of C-MWCNT because of the introduction of amine groups onto carboxyl groups. In addition, there was only a small reduction of peaks after lead adsorption compared with C-MWCNT, indicating that carboxyl-lead complexes did not dominate the sorption capacity for N-MWCNT (Fig 2(C) and 2(D)). However, it was confirmed that the peak of CONH (402.3 eV) reduced after lead adsorption onto the N-MWCNT, which suggested the formation of amide-lead complexes [27, 29]. On the basis of the XPS results, the mechanism for lead adsorption onto F-MWCNTs is illustrated in Fig 3.

### Effect of F-MWCNTs on lead toxicity

The effect of F-MWCNTs on lead toxicity was investigated by comparing the acute toxicity of lead before and after adsorption onto F-MWCNTs. The 48 h LC_50_ values of C-MWCNT and N-MWCNT were 53.3 and 40.0 mg L^-1^, respectively (S4 Fig). Mortality of daphnids was not observed when they were exposed to up to 20 mg L^-1^ of F-MWCNTs; therefore, we performed the toxicity test with the concentrations of F-MWCNTs below 20 mg L^-1^, which simplified our experiment on the effect of F-MWCNTs on the toxicity and bioavailability of lead. Previous studies showed that residual metal impurities in MWCNTs could enhance the toxic effects of MWCNTs as well as other toxic materials (e.g., Cd) [30, 31]. However, in this study the toxic effect of metal impurities in F-MWCNTs was negligible in comparison with the acute toxicity of metals to *D*. *manga* (S1 Table).

**Fig 4:**
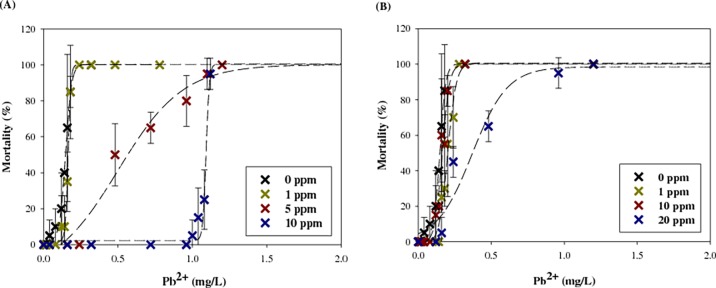
Effect of (A) C-MWCNT and (B) N-MWCNT on lead toxicity to *D. magna*. Error bars represent the standard deviation.

The toxicity of lead to *D*. *magna* in the presence or absent of F-MWCNTs is shown in Fig 4 and Table 2. In the presence of 1 mg L^-1^ C-MWCNT, the LC_50_ value of lead showed no obvious differences. However, the adverse effect of lead was substantially alleviated at concentrations of C-MWCNT above 5 mg L^-1^. In the case of N-MWCNT, lead toxicity showed no changes at concentrations up to 10 mg L^-1^ of N-MWCNT, but it lessened at 20 mg L^-1^. The reduced toxic effect of lead is likely to be related with the adsorption of lead onto the F-MWCNTs. The reduced toxicity is consistent with previous findings that functionalized multi-walled carbon nanotubes decreased cadmium toxicity in *D*. *magna*, although F-CNTs of different lengths had different effects on toxicity [31]. According to the study, reduced cadmium toxicity was due to decreased cadmium bioaccumulation. Ingestion of CNTs influenced the physiological activity of *D*. *magna* and subsequently inhibited cadmium uptake.

**Table 2:** Effect of C-MWCNT and N-MWCNT on LC_50_ (48 h) values and their 95% confidence intervals (CI) of lead.

	LC_50_ (95% CI) (Pb^2+^ mg L^-1^)	
Conc. of F-MWCNTs	C-MWCNT	N-MWCNT
0 mg L^-1^	0.15 (0.13–0.16)	0.15 (0.13–0.16)
1 mg L^-1^	0.17 (0.15–0.17)	0.20 (0.18–0.24)
5 mg L^-1^	0.55 (0.47–0.63)	-^1)^
10 mg L^-1^	1.08 (1.08–1.08)	0.16 (0.15–0.20)
20 mg L^-1^	-^1)^	0.33 (0.27–0.40)

^1)^ Experiment was not carried out in this study

### Effect of F-MWCNTs on bioaccumulation of lead

**Fig 5:**
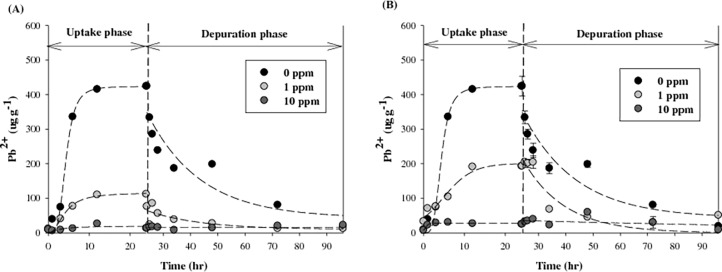
Effect of (A) C-MWCNT and (B) N-MWCNT on bioaccumulation of lead in *D. magna*. Error bars represent the standard deviation.

For further examination of the mechanisms behind the alleviation of lead toxicity by the F-MWCNTs, bioaccumulation of lead was quantified with or without F-MWCNTs. Results for accumulated lead in *D*. *magna* after exposure to 10 μg L^-1^ of lead (Fig 5) show that, when *D*. *magna* was exposed to lead only, lead concentration in the body increased quickly within 12 h and slowly reached equilibrium (424.3± 28.2 μg g^-1^) after 24 h; however, in the presence of F-MWCNTs, a notable difference was observed in the amount of bioaccumulated lead. Especially, the addition of 10 mg L^-1^ of F-MWCNTs resulted in a large reduction in lead accumulation in *D*. *magna*. The maximum concentrations of lead in daphnids in the presence of 10 mg L^-1^ of C-MWCNT and N-MWCNT were 13.6±0.2 and 25.3±0.9 μg g^-1^, respectively. The effect of C-MWCNTs on lead accumulation was much higher than that of N-MWNCTs, which is a similar result to that of the acute toxicity test.

The elimination kinetics of lead from *D*. *magna* are also shown in Fig 5. The lead accumulated in daphnids was rapidly eliminated during the depuration period. In the case of lead exposed with F-MWCNTs, the lead concentration in daphnids also decreased with time, but with smaller elimination rate constants and longer half-lives compared with lead exposed alone (Table 3). These results suggest that lead accumulated in daphnids may be retained for a long time when lead is exposed with F-MWCNTs.

**Table 3:** Elimination kinetic indices of lead in *D*. *magna*.

Exposure condition	*K*_*e*_ (h^-1^)	*t*_1/2_ (h)
Lead	0.036	19.0
Lead exposed with 1 ppm C-MWCNT	0.023	29.8
Lead exposed with 10 ppm C-MWCNT	-^1)^	- ^1)^
Lead exposed with 1 ppm N-MWCNT	0.024	28.9
Lead exposed with 10 ppm N-MWCNT	0.013	54.6

^1)^ Concentration of lead in *Daphnia magna* was extremely low; therefore, toxicokinetic indices were not evaluated.

## Discussion & conclusion

The effects of two different functionalized carbon nanotubes on lead toxicity and bioaccumulation were investigated with a freshwater zooplankton, *Daphnia magna*. The acute toxicity results indicate that different surface properties of MWCNTs perform differently in affecting lead toxicity to *D*. *magna*. Carboxylate modified MWCNT (C-MWCNT) showed a large decrease in lead toxicity, whereas polyethyleneimine modified MWCNT (N-MWCNT) had only a slight effect on lead toxicity. The different toxicity results seem to be connected with the adsorption properties of the F-MWCNTs. According to previous studies, adsorption of metal pollutants from water is highly dependent on the surface properties of adsorbents [14, 32, 33]. In addition, it is suggested that metal adsorption onto functionalized nanoparticles is via both electrostatic interaction and formation of strong coordinate bonds between the metals and functionalized groups of nanoparticles [6, 34, 35]. In this study, the adsorption capacity of C-MWCNT was much higher than that of N-MWNCT, indicating the preferential electrostatic interaction between negatively charged C-MWCNT and lead ions, Pb^2+^. The EPM results indirectly revealed the electrostatic interaction between F-MWCNT and lead ions during the adsorption experiment. After lead adsorption, the EPM values of C-MWCNT slightly shifted to positive charge, but those of N-MWCNT rarely changed (S2 Fig). The results are consistent with previous findings that carboxylate functionalized single-walled carbon nanotubes (SWCNTs) can more effectively adsorb heavy metals than pristine SWCNT because of the carboxyl group, which increases negative charge on the carbon nanotube surface [36]. The electrostatic interactions between metal ions and surface charge of MWCNTs were proposed as the predominant mechanism of metal adsorption onto F-MWCNTs [6]. However, the electrostatic interactions mainly contribute at low pH and are relatively weak interactions. The strong adsorption of metals on the MWCNTs arises from chemical interaction between metal ions and the surface sites of carbon nanotubes. In this study, two different functional groups were employed to compare the maximum adsorption capacities. In comparison with N-MWCNT, C-MWCNT showed a high adsorption capacity, suggesting that the carboxyl-lead complex is much stronger than the amide-lead complex. In addition, the lower content of acidic functional groups (-COOH) in N-MWCNT could account for the lower adsorption affinities.

The physicochemical properties of F-MWCNTs showed notable alteration after lead adsorption. Although F-MWCNTs were highly stable in experimental solutions, these particles agglomerated together (S1 Fig) and settled out (S5 Fig) after lead adsorption. These results suggest that F-MWCNTs do not act as a carrier but rather as a scavenger for toxic lead ions, at least in our experimental conditions. Therefore, it could be predicted that lead toxicity in the real environment is alleviated when F-MWCNTs enter aquatic environments; however, there are still diverse opinions concerning the effects of CNTs on hazardous materials. Wang et al. [37] showed increased Cd toxicity in *D*. *magna* under F-MWCNT exposure. Also Kim et al. [38] demonstrated that CNTs could enhance Cu toxicity in the presence of natural organic matters. In contrast, Liu et al. [32] showed reduced Cd toxicity under CNT exposure and suggested that different physicochemical properties of CNTs could affect the metal accumulation in *D*. *magna*. It is well known that the adsorption of heavy metals is dependent on the surface properties of nanoparticles as well as environmental conditions such as pH, ionic strength, concentration of pollutants, and amount of adsorbents [6, 7, 22]. Furthermore, the physicochemical properties of nanoparticles could be altered by environmental conditions [39, 40]. Therefore, it is suggested from the present study that the interaction of heavy metals and nanoparticles should be considered carefully in any prediction of environmental risk assessment of F-MWCNTs.

## Supporting information

S1 Table Concentrations of metal impurities and acute toxicity to *D*. *magna*. (PDF)

S1 Fig Transmission electron microscopy (TEM) images of F-MWCNTs. (TIF)

S2 Fig Electrophoretic mobilities (EPM) of F-MWCNTs. (TIF)

S3 Fig XPS wide-scan of F-MWCNTs. (TIF)

S4 Fig Mortality of *D*. *magna* exposed to various concentrations of F-MWCNTs. (TIF)

S5 Fig Sedimentation kinetics of F-MWCNTs (5mg/L) with various concentrations of lead. (TIF)
