# Supplementary material for: Effects of functionalized multi-walled carbon nanotubes on toxicity and bioaccumulation of lead in Daphnia magna
Source: PLoS One. 2018 Mar 29;13(3):e0194935. doi: 10.1371/journal.pone.0194935 (PMC5875790; doi:10.1371/journal.pone.0194935)
Supplement: S1 Table — (PDF) [file pone.0194935.s001.pdf]

S1 Table. Concentrations of metal impurities and acute toxic values to *D. magna*.

Metal impurities	Metal concentrations				Acute toxicity of metals to *D. magna* [48h-EC_{50}] (ug/L)[c]
	Metal conc. in F-MWCNTs (ug/g) [a]		Metal conc. in M4 media (ug/L) [b]		
	C-MWCNT	N-MWCNT	C-MWCNT	N-MWCNT	
Cr	8.28	1.31	0.21	0.03	130
Co	N.D.	N.D.	-	-	710
Ni	4.88	N.D	0.12	-	650
Mo	122.13	507.79	3.05	12.69	1,500,000
Pb	0.37	0.81	0.01	0.02	290
Cu	N.D	N.D	-	-	13
Cd	N.D	N.D	-	-	3.6
Zn	6.82	3.07	0.17	0.08	720
Al	86.56	33.87	2.16	0.85	3,900
Fe	93.95	81.49	2.35	2.04	2,300
Y	93.95	81.49	2.35	2.04	490

[a] *Metal concentrations in F-MWCNTs were measured by ICP/MS after aqua regia digestion.*
[b] *Metal concentrations in M4 media (test media) were calculated on the assumption that 20 mg L^{-1} F-MWCNTs were exposed to D.magna in 40 mL of test media..*
[c] *Acute toxicity of metals to D. magna (EC_{50} values) was quoted from a research article studied by Okamoto et al [1].*

Reference

1. Okamoto A, Yamamuro M, Tatarazako N. Acute toxicity of 50 metals to Daphnia magna. J. Appl. Toxicol. 2015;35:824–30.
