# Supplementary figures and images for: Effects of functionalized multi-walled carbon nanotubes on toxicity and bioaccumulation of lead in Daphnia magna
Source: PLoS One. 2018 Mar 29;13(3):e0194935. doi: 10.1371/journal.pone.0194935 (PMC5875790; doi:10.1371/journal.pone.0194935)

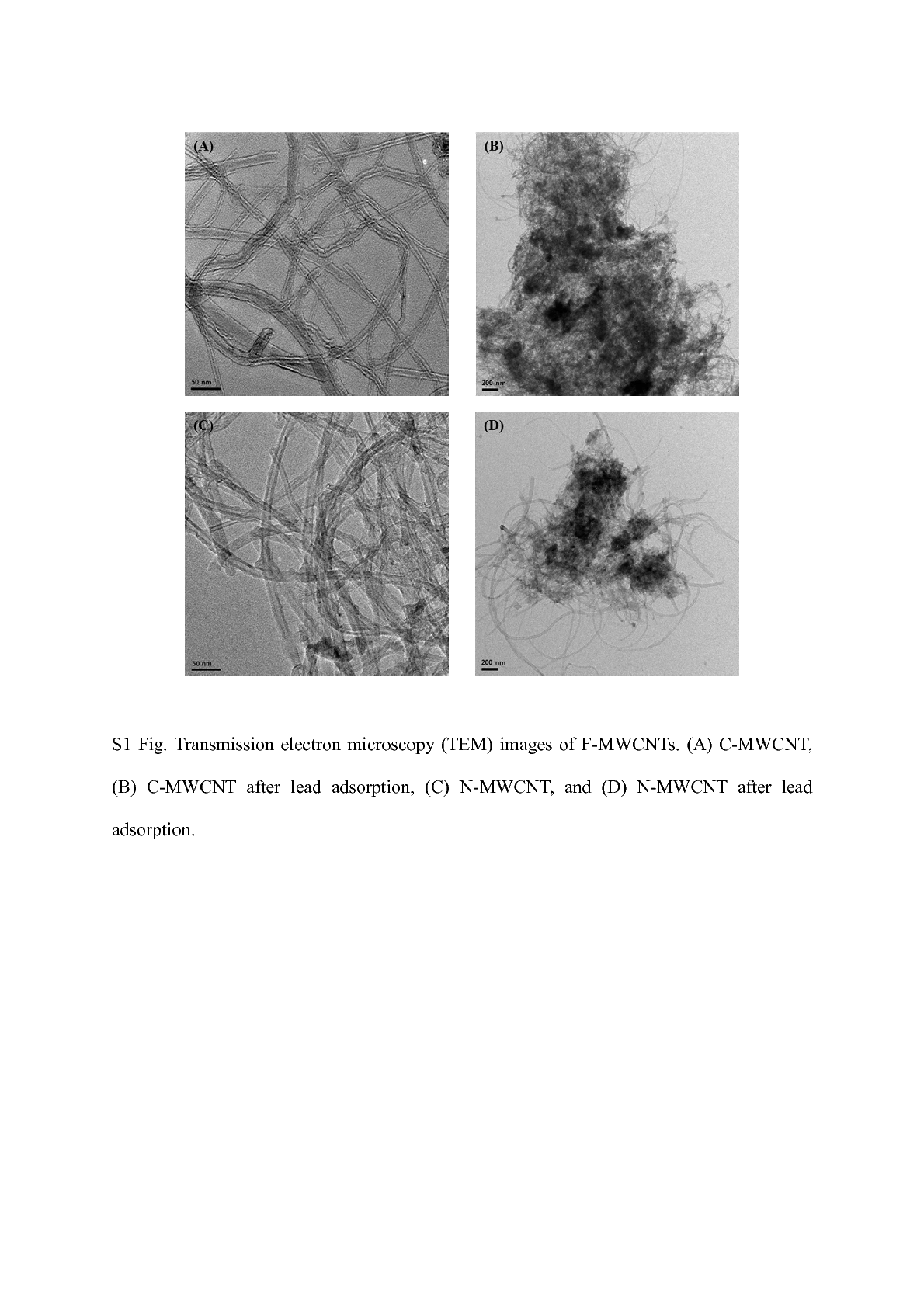

Supplement: S1 Fig — (TIF) [file pone.0194935.s002.tif]

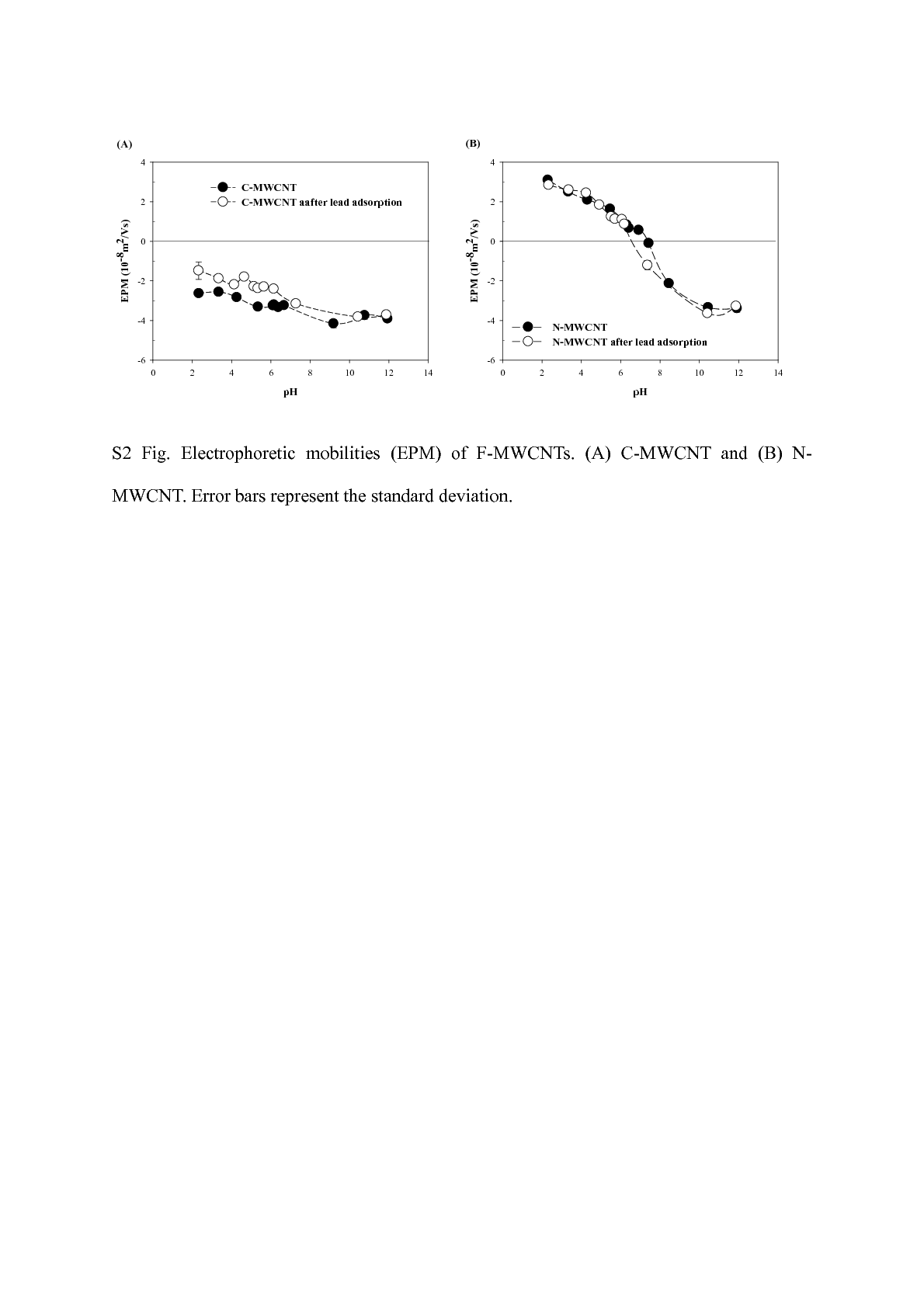

Supplement: S2 Fig — (TIF) [file pone.0194935.s003.tif]

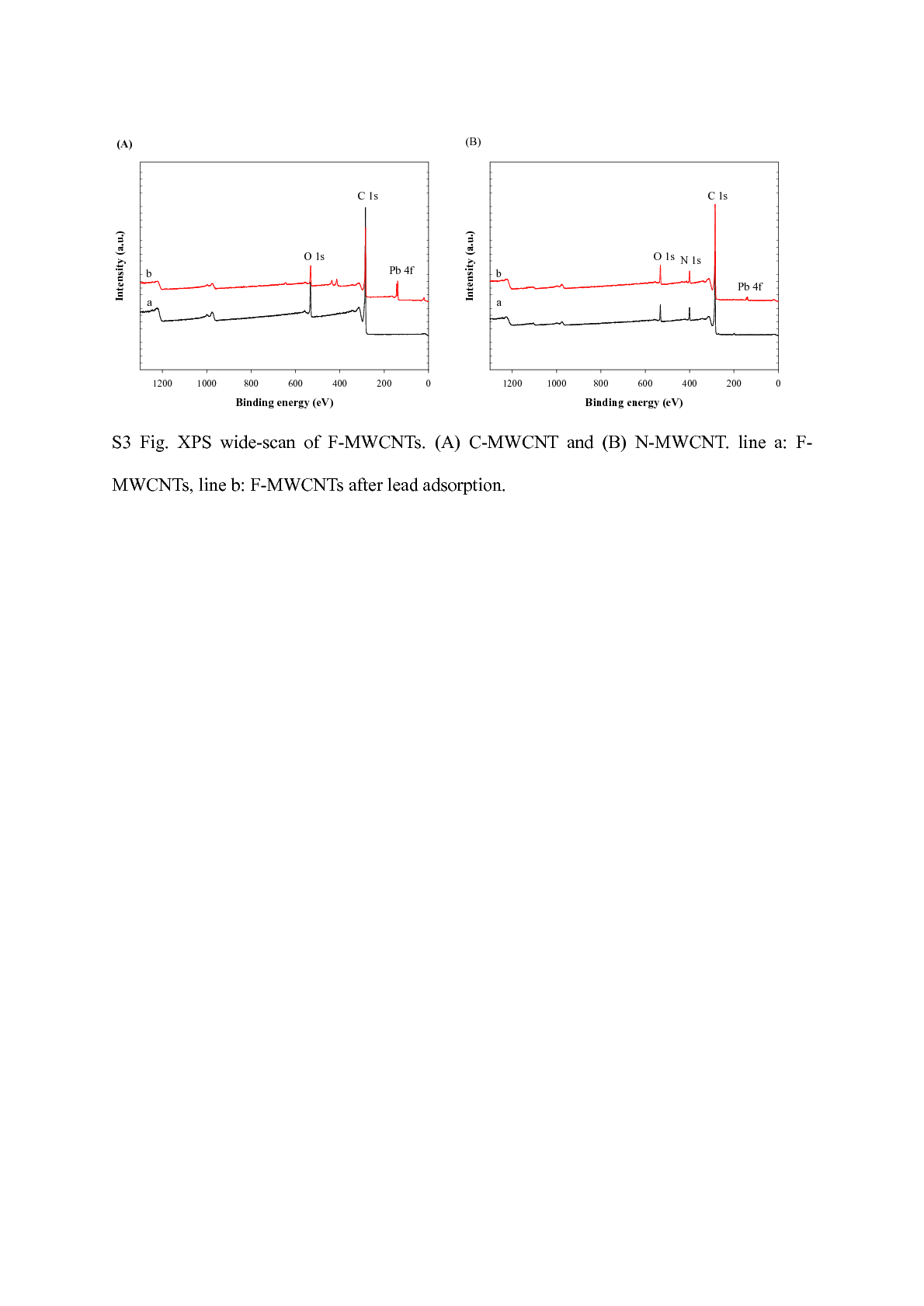

Supplement: S3 Fig — (TIF) [file pone.0194935.s004.tif]

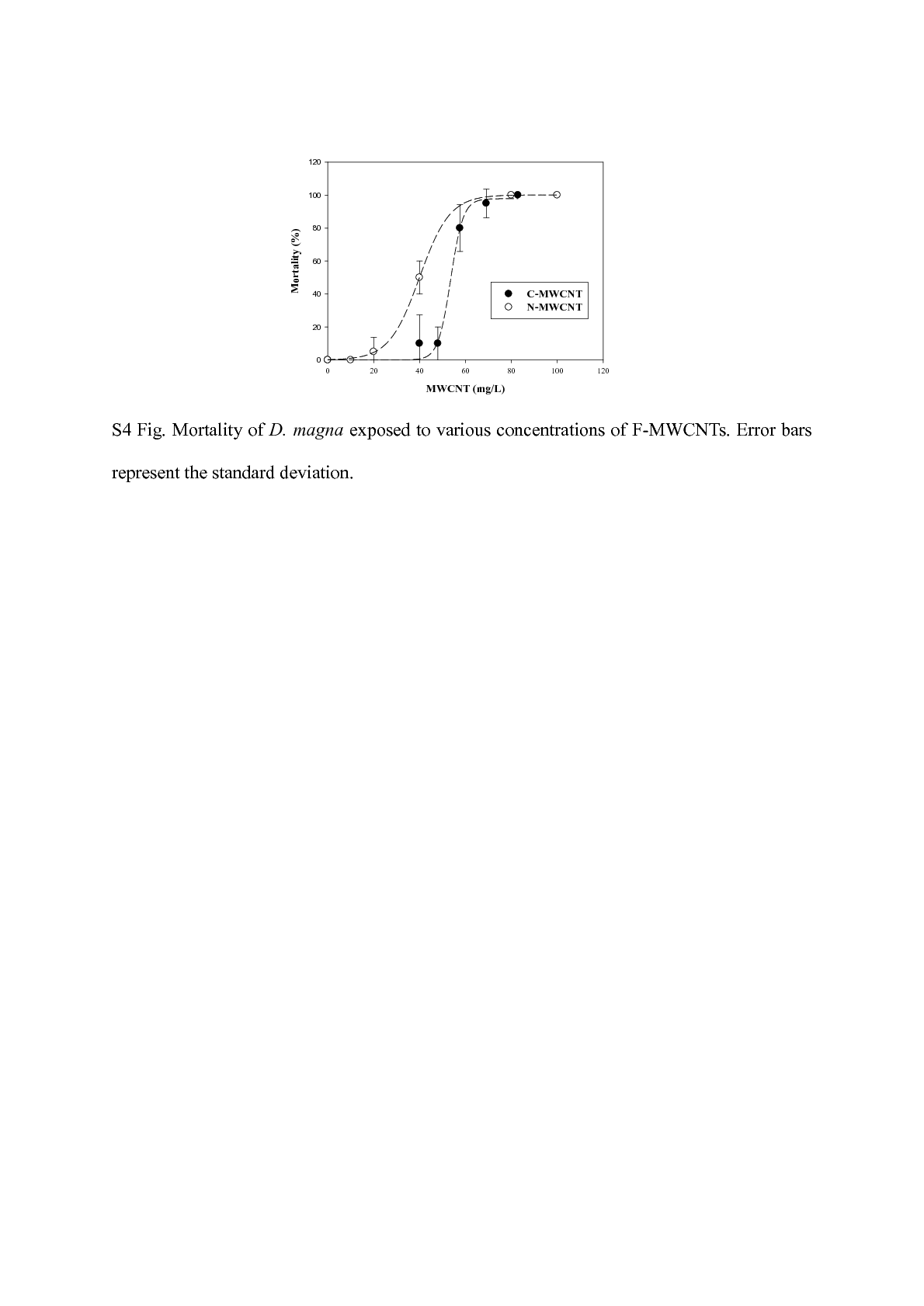

Supplement: S4 Fig — (TIF) [file pone.0194935.s005.tif]

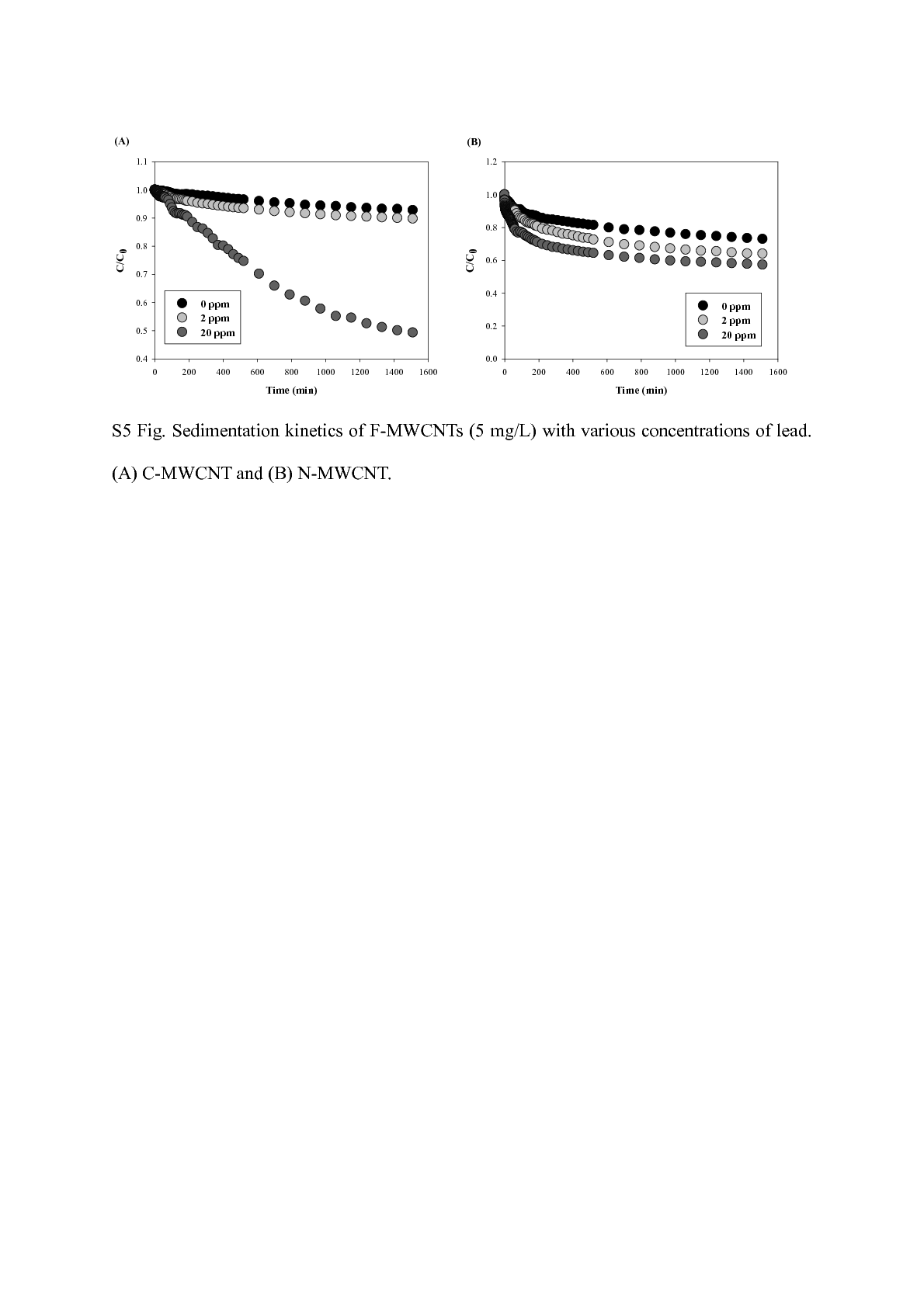

Supplement: S5 Fig — (TIF) [file pone.0194935.s006.tif]
